# Draft genome sequences of *Shewanella* spp. isolated from the marine sediment in British Columbia, Canada

**DOI:** 10.1128/mra.00976-25

**Published:** 2025-12-23

**Authors:** Xiaoji Liu, Scott Hrycauk, Mruthula Narayan, Skyler Umlah

**Affiliations:** 1 Agriculture and Agri-Food Canada, Lacombe Research and Development Centre 98668, Lacombe, Alberta, Canada; 2 Faculty of Science, University of British Columbia 8166, Vancouver, BC, Canada; 3 Faculty of Science, Red Deer Polytechnic 6827 , Red Deer, Alberta, Canada; Montana State University, Bozeman, Montana, USA

**Keywords:** Atlantic salmon, sediment, *Shewanella*, bioremediation

## Abstract

We present the draft genome sequences of 32 *Shewanella* spp. isolated from the marine sediment in British Columbia, Canada. The sediment was collected from and near a fallowed Atlantic salmon (*Salmo salar*) farm; thus, our research highlights the uniqueness of *Shewanella* spp. associated with the region.

## ANNOUNCEMENT

*Shewanella* spp. are important environmental microorganisms in degrading organic matters and recycling of carbon and metals supporting marine ecosystems (1–3). However, *Shewanella* spp. from marine sediment in British Columbia, Canada have not been documented. Here, we have isolated and sequenced the whole genomes of *Shewanella* spp. from the marine sediment in Phillips Arm (55.6°N, 125.4°W). The sediment was collected from a historical salmon farming site as part of routine monitoring sampling from the aquaculture monitoring program by Fisheries and Oceans Canada (DFO). Sampling locations included directly beneath the salmon cage (0 m distance, 85 m depth), 30 m distance (80 m depth), and 125 m distance (76 m depth) along the water current from the cage site.

Bacteria were isolated by streaking the sediment on tryptic soy agar (TSA, MP Biomedicals, CA) supplemented with 2% NaCl and 1 µg/mL tetracycline cultured at 15°C aerobically. A single colony was transferred to 10 mL tryptic soy broth (TSB) with 2% NaCl and cultured as above. Cell pellets were obtained by centrifugation at 5,000 ×*g* for 10 min at 22°C. Total DNA was extracted using DNeasy Blood & Tissue Kit (Qiagen, Canada) with modification of pretreatment of cell pellets with enzymatic lysis buffer as specified in the protocol for bacterial DNA extraction at 22°C for 1.5 h.

Total DNA was subjected to whole-genome sequencing (WGS) at Génome Québec on the Illumina NovaSeq X Plus sequencing system (150 bp paired end) using the NEBNext Ultra II DNA Library Prep Kit (New England BioLabs). Quality control of Illumina reads was performed by fastp (version 0.23.2; [4]) with Phred score cut-off of 15. Genome was assembled using SPAdes with basic required option (version 3.15.5; [5]), and the quality and completeness of genome assembly were checked by QUAST (version 4.6.3; [6]). Taxonomic classification was performed by GTDB-Tk (version 2.3.2 classify_wf; (7)). Default parameters were used, except where otherwise noted. All of the bioinformatic analyses were performed on the PHAC-NML’s high-performance computing cluster (Waffles), as in our previous study (8).

Phylogenetic analysis was conducted on the genome sequences of *Shewanella* spp. on Type Strain Genome Server (9, 10) without outgroup using default selection of representative type strain genomes, including *S. japonica* KCTC 22435, a well-known *Shewanella* from the Pacific region (11). The maximum-likelihood phylogenetic tree was visualized with iTOL (12). Annotation genome completeness of the genome was carried out by National Center for Biotechnology Information’s PGAP (version 6.10; [13]) and CheckM (version 1.2.4; [14]).

**Fig 1:**
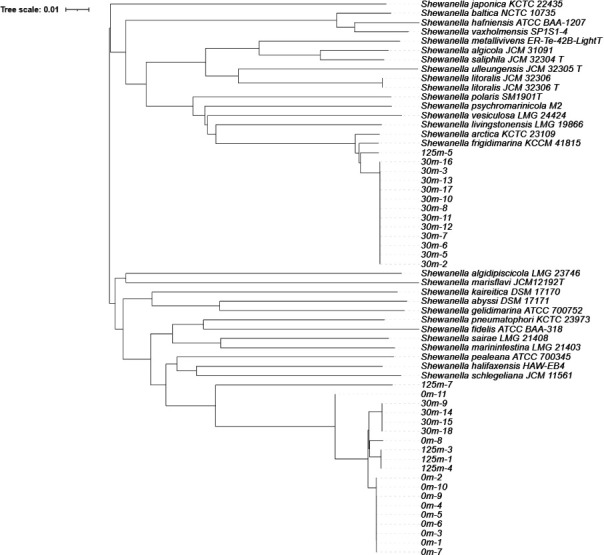
Phylogenetic tree based on WGS constructed in Type Strain Genome Server showing the *Shewanella* from marine sediment in British Columbia, Canada. Average brunch support: 47.2%. Delta statistics: 0.12.

An average of 34,771,224 reads per strain ≥ quality of 39 was obtained with completeness >99.5%. The average genome size of these *Shewanella* spp. is 4.79 Mb with an average GC content of 42.95%. The raw reads, genome size, GC%, contigs, N50, genes, and protein coding genes are summarized in Table 1. Phylogenetic analysis showed that the *Shewanella* spp. from our study were not closely related to *S. japonica* KCTC 22435 (Fig. 1). Those isolated from 30 m mostly were closely related to *S. frigidimarina* KCCM 41815, which was isolated from Antarctica (assembly accession: GCF_003797845.1/).

**TABLE 1:** Genome information of *Shewanella* spp. from marine sediment in BC, Canada

Strain	Raw reads	Genome size (Mb)	GC (%)	Contigs	Scaffold N50 (kb)	Total genes	Protein-coding genes	Assembly accession	Raw reads accession
0m-1	37,652,687	4.8	44	79	139.1	4,090	3,984	GCA_052977305.1/	SRX30001699[accn]
0m-2	34,616,151	4.8	44	81	131.5	4,091	3,984	GCA_052977385.1/	SRX30001700[accn]
0m-3	35,016,488	4.8	44	80	138.9	4,079	3,984	GCA_052977365.1/	SRX30001702[accn]
0m-4	35,717,795	4.8	44	79	139.1	4,088	3,982	GCA_052977005.1/	SRX30001703[accn]
0m-5	37,944,752	4.8	44	79	139.2	4,085	3,980	GCA_052977145.1/	SRX30001709[accn]
0m-6	35,446,935	4.8	44	76	142.8	4,085	3,982	GCA_052977325.1/	SRX30001708[accn]
0m-7	38,225,725	4.8	44	77	139.2	4,084	3,982	GCA_052977285.1/	SRX30001707[accn]
0m-8	20,137,083	5	43.5	117	83.9	4,320	4,198	GCA_052977265.1/	SRX30001706[accn]
0m-9	34,326,323	4.8	44	76	138.8	4,088	3,990	GCA_052977085.1/	SRX30001705[accn]
0m-10	30,793,556	4.8	44	78	138.9	4,080	3,983	GCA_052977225.1/	SRX30001704[accn]
0m-11	32,905,687	4.7	43.5	4059	1.2	6,446	6,372	GCA_052977245.1/	SRX30001701[accn]
30m-2	36,130,083	4.7	41.5	53	196	4,069	3,979	GCA_052172295.1/	SRX29978595[accn]
30m-3	31,816,683	4.7	41.5	54	207.1	4,082	3,981	GCA_052172555.1/	SRX29978596[accn]
30m-5	30,914,244	4.7	41.5	55	225	4,080	3,980	GCA_052172355.1/	SRX29978600[accn]
30m-6	36,263,240	4.9	41.5	383	184.6	4,455	4,347	GCA_052172415.1/	SRX29978601[accn]
30m-7	37,792,858	4.7	41.5	54	207.1	4,068	3,980	GCA_052172455.1/	SRX29978602[accn]
30m-8	38,090,930	4.7	41.5	56	204	4,082	3,982	GCA_052172515.1/	SRX29978603[accn]
30m-9	37,428,727	5.1	44	437	147.9	4,531	4,428	GCA_052977025.1/	SRX29989771[accn]
30m-10	40,870,887	4.7	41.5	53	230.5	4,080	3,981	GCA_052172535.1/	SRX29978604[accn]
30m-11	35,410,592	4.7	41.5	63	189.8	4,084	3,988	GCA_052172475.1/	SRX29978605[accn]
30m-12	36,486,497	4.7	41.5	53	207.1	4,080	3,979	GCA_052172275.1/	SRX29978606[accn]
30m-13	29,681,013	4.7	41.5	58	196	4,075	3,981	GCA_052172495.1/	SRX29978607[accn]
30m-14	35,343,216	4.8	44	64	148.2	4,099	4,007	GCA_052977125.1/	SRX29989772[accn]
30m-15	37,393,034	4.8	44	65	148.2	4,108	4,009	GCA_052977185.1/	SRX29989773[accn]
30m-16	26,485,992	4.7	41.5	62	207.1	4,089	3,986	GCA_052172395.1/	SRX29978597[accn]
30m-17	39,367,279	4.7	41.5	53	230.5	4,083	3,981	GCA_052172375.1/	SRX29978598[accn]
30m-18	34,516,155	4.8	44	63	157.4	4,102	4,008	GCA_052977205.1/	SRX30291147[accn]
125m-1	29,276,410	4.9	44	106	82.7	4,140	4,045	GCA_052977065.1/	SRX29989774[accn]
125m-3	33,611,949	4.9	44	110	82.7	4,154	4,047	GCA_052977105.1/	SRX29989778[accn]
125m-4	37,906,781	4.9	44	110	78.1	4,140	4,043	GCA_052977165.1/	SRX29989777[accn]
125m-5	34,845,715	4.7	41.5	46	219.4	4,054	3,968	GCA_052172435.1/	SRX29978599[accn]
125m-7	40,263,686	4.8	44	113	83.4	4,134	4,009	GCA_052977045.1/	SRX29989776[accn]

## Data Availability

This Whole Genome Shotgun project has been deposited in Sequence Read Archive (SRA) under BioProject PRJNA1298336. The genome assembly and raw reads accession no. are listed in Table 1. The version described in this paper is the first version of each strain.
